# FAM65A, as a potential predictor of prognosis, promotes colorectal cancer progression via activating Ras/ERK/RSK signaling

**DOI:** 10.1016/j.isci.2026.114662

**Published:** 2026-01-10

**Authors:** Yuqiu Ma, Jie Yao, Xinzhuang Shen, Shuying Wang, Gongli Tang, Xiaowen Yang, Yifei Li, Yifang Sun, Wenzhi Shen, Xiaoyuan Zhang, Yongming Huang

**Affiliations:** 1 Department of General Surgery, Affiliated Hospital of Jining Medical University, Jining Medical University, Jining 272000, China; 2 Key Laboratory of Precision Oncology in Universities of Shandong, Institute of Precision Medicine, Jining Medical University, Jining 272067, China; 3 Henan Key Laboratory of Immunology and Targeted Drugs, School of Laboratory Medicine, Xinxiang Medical University, Xinxiang 453003, China; 4 Department of Oncology, Jining Hospital of Traditional Chinese Medicine, Jining 272000, China; 5 Local Town Centre Health Centre of Difang, Pingyi County, Shandong Province 273306, China; 6 Department of Ultrasound, Affiliated Hospital of Jining Medical University, Jining 272000, China

**Keywords:** Molecular biology, Cell biology, Cancer

## Abstract

Research indicates that FAM65A is significantly involved in tumorigenesis. Nevertheless, the prognostic implications of FAM65A expression levels and its contribution to CRC malignant progression have yet to be elucidated. Here, we revealed that *FAM65A* is overexpressed in CRC tissues and is linked to various pathological indicators and patient prognosis. Importantly, Cox regression analysis indicated that *FAM65A* may function as an independent prognostic marker. Furthermore, functional assays conducted *in vitro* demonstrated that FAM65A enhanced CRC cell proliferation and migration, alongside decreased apoptosis. Mechanistically, we elucidated that FAM65A binds to Ras and activates the Ras/extracellular regulated protein kinases (ERK) signaling to mediate RSK activation contributes to CRC progression, treatment with the Ras inhibitor Abd-7 or RSK inhibitor BRD7389 effectively countered the FAM65A-mediated enhancement of malignancy. Additionally, *in vivo* experiments indicated that FAM65A knockdown led to the inhibition of Ras/ERK/RSK activation and subsequently impeded CRC progression. Our study provides targets and strategies for the treatment of CRC.

## Highlights

• FAM65A is overexpressed in CRC tissues and correlates with pathological features
• FAM65A may serve as an independent prognostic biomarker for CRC patients
• FAM65A expression promotes CRC malignant progression *in vitro* and *in vivo*
• FAM65A binds to Ras and activates Ras/ERK/RSK signaling to mediate CRC progression

## Graphical abstract

**Figure:**
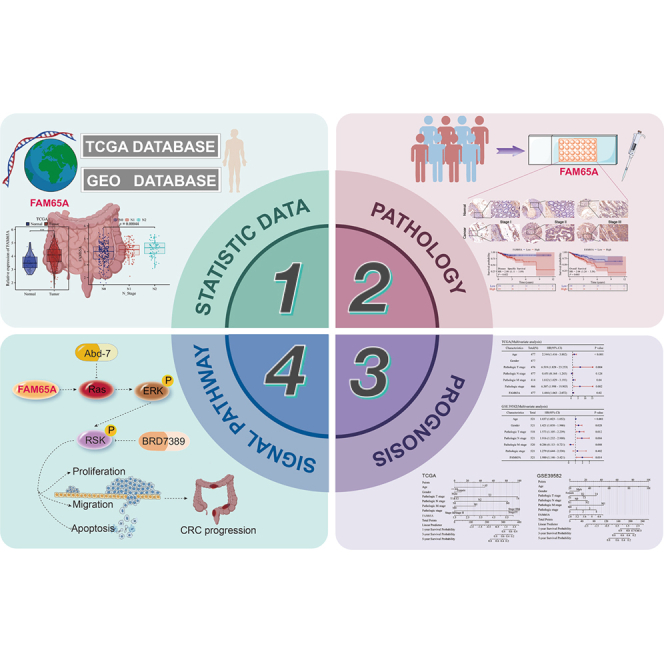

## Introduction

Colorectal cancer (CRC) is one of the most prevalent malignancies among humans, exhibiting a high incidence and mortality rate, and it ranks third in terms of diagnoses among men and second among women.^1^^,^^2^ Despite significant advancements in screening and treatment methodologies, which have led to a marked improvement in prognosis and a notable increase in the 5-year relative survival rate,^3^ challenges remain. Effective strategies for preventing the onset of CRC, particularly those aimed at addressing tumor metastasis, chemoresistance, and recurrence remain insufficient. Consequently, the identification of biomarkers for the early detection of CRC is an urgent concern in the ongoing efforts to enhance treatment outcomes for this disease.

Rho guanosine triphosphate (GTP)ases exist in two states: an inactive form bound to guanosine diphosphate (GDP) and an active form bound to GTP.^4^ These proteins are regulated by numerous effector proteins that are crucial for a variety of biological functions.^5^^,^^6^ Recent research has indicated that Rho GTPases exhibit dysregulation in multiple cancer types and play a multifaceted role in tumor progression.^7^^,^^8^^,^^9^ Family with sequence similarity 65 member A (*FAM65A*), also referred to as *RIPOR1*, is a newly identified effector of the RHO subfamily of Rho GTPases (Ras homolog family).^10^ FAM65A interacts with GTP-bound RHO proteins (specifically Ras homolog family member A [RHOA], Ras homolog family member B [RHOB], and Ras homolog family member C [RHOC]) via its N-terminal HR1 structural domain.^11^ It is significant in the regulation of cell migration polarity mediated by RHO, particularly through the reorientation of the Golgi apparatus.^11^ In addition, it has been reported that FAM65A protein may be a biomarker for patients with cholangiocarcinoma.^12^ Nevertheless, there is a paucity of research regarding the functional implications of FAM65A, particularly concerning its relationship with CRC.

Protein synthesis represents a critical process during the G0/G1 and G2/M phases of the cell cycle.^13^ The ribosomal S6 protein kinase (RSK) family, which comprises four principal isoforms (RSK1, RSK2, RSK3, and RSK4), functions downstream of the mitogen-activated protein kinase (MAPK) signaling pathway.^14^^,^^15^ Research has demonstrated that RSKs play diverse roles, particularly in the proliferation, invasion, and migration of cancer cells.^16^^,^^17^^,^^18^^,^^19^^,^^20^ Furthermore, the RSK family is implicated in the regulation of the tumor microenvironment by influencing integrin-mediated cell adhesion and cytoskeletal dynamics.^21^ Nevertheless, the specific role of RSK in the progression of CRC mediated by FAM65A remains to be investigated.

In this study, we aim to examine the function of FAM65A in CRC and its association with various pathological markers and patient outcomes. Our analysis will focus on the impact of FAM65A expression on CRC cell proliferation, apoptosis, and migration, as well as its influence on the malignant progression of CRC in murine models. Furthermore, we seek to elucidate the molecular mechanisms by which FAM65A promotes malignant progression in CRC through binding to Ras and activating the Ras/extracellular regulated protein kinases (ERK) signaling pathway to mediate RSK activation. It may yield therapeutic targets and strategies for the management of CRC.

## Results

### FAM65A was highly expressed in CRC tissues

**Figure 1:**
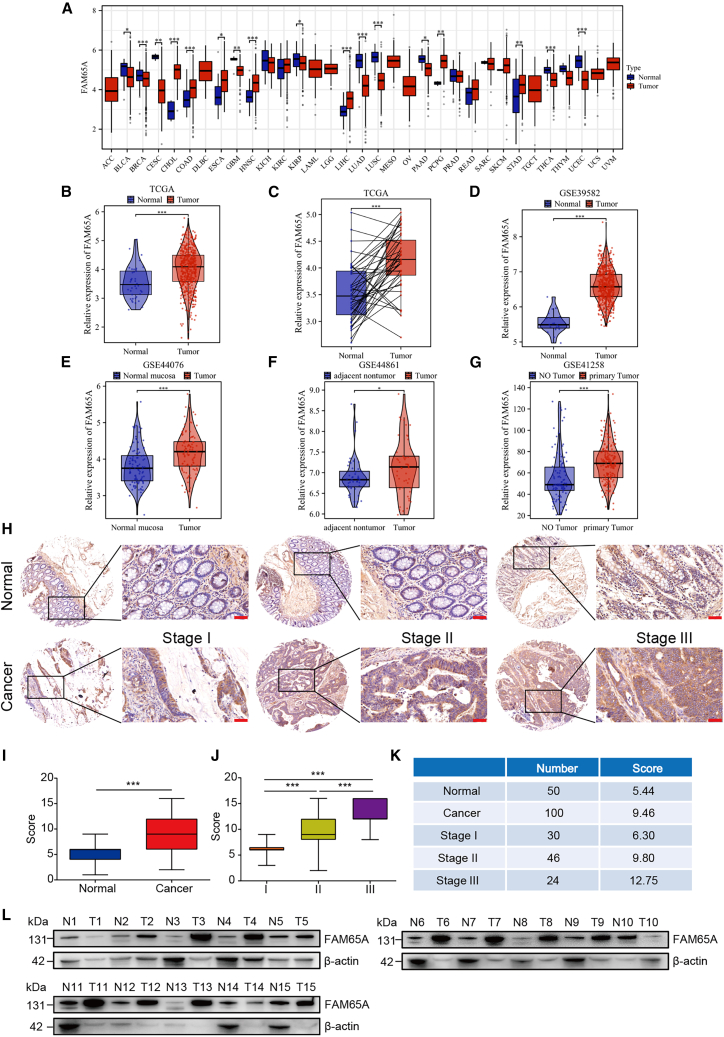
FAM65A was highly expressed in CRC tissue (A) The expression of *FAM65A* across various cancer types as documented in the TCGA database. (B) Comparative analysis of *FAM65A* expression levels in 480 colon cancer specimens and 41 normal tissue samples from the TCGA database. ∗∗∗*p* < 0.001. (C) Assessment of *FAM65A* expression in 41 colon cancer samples alongside 41 matched normal tissues. ∗∗∗*p* < 0.001. (D–G) Analysis of *FAM65A* expression levels utilizing the GEO dataset GEO: GSE39582 (N = 19, T = 566), GSE44076 (N = 98, T = 98), GSE44861 (N = 46, T = 49), GSE41258 (N = 102, T = 186). ∗*p* < 0.05, ∗∗∗*p* < 0.001. (H) IHC findings pertaining to normal and colon cancer tissues, including TNM staging. Scale bars, 50 μm. (I–K) Quantitative evaluation of FAM65A expression in normal versus colon cancer tissues, incorporating TNM staging data. ∗∗∗*p* < 0.001. (L) Western blot analysis illustrating FAM65A expression in colon cancer tissues compared to adjacent normal tissues; “N” denotes normal and “T” denotes tumor. Data are presented as mean ± SEM.

To explore the possible role of *FAM65A* in tumorigenesis, we analyzed the expression levels of *FAM65A* in tumor and normal tissue samples from multiple cancer types in the TCGA (The Cancer Genome Atlas) database. The results revealed that *FAM65A* was highly expressed in seven tumors including colon adenocarcinoma (Figure 1A). Further data analysis results demonstrated that *FAM65A* expression was higher in matched colon cancer tissues than in adjacent samples (Figures 1B and 1C). In addition, the expression results of *FAM65A* in multiple GEO (Gene Expression Omnibus) datasets GEO: GSE39582, GSE44076, GSE44861, and GSE41258 were the same as those in TCGA datasets (Figures 1D–1G).

To corroborate these findings, we conducted immunohistochemistry (IHC) utilizing a human tissue microarray. The results indicated that *FAM65A* was significantly overexpressed in colorectal tissues when compared to normal tissues (Figures 1H and 1I). Additionally, we explored the association between *FAM65A* expression and tumor tumor node metastasis (TNM) staging, revealing a positive correlation between *FAM65A* levels and the TNM stage of the tumors (Figures 1J and 1K). For further validation, proteins were extracted from 15 cases of adjacent non-cancerous and CRC tissues for western blot analysis. The results demonstrated that *FAM65A* was highly expressed in 13 out of the 15 CRC samples relative to the adjacent non-cancerous tissues, although two cases exhibited slightly reduced expression levels of *FAM65A* (Figures 1L and S1A). Therefore, it can be concluded that *FAM65A* is significantly overexpressed in human CRC tissues in comparison to normal tissues.

### *FAM65A* expression was correlated with the clinicopathological features and prognosis of patients

**Figure 2:**
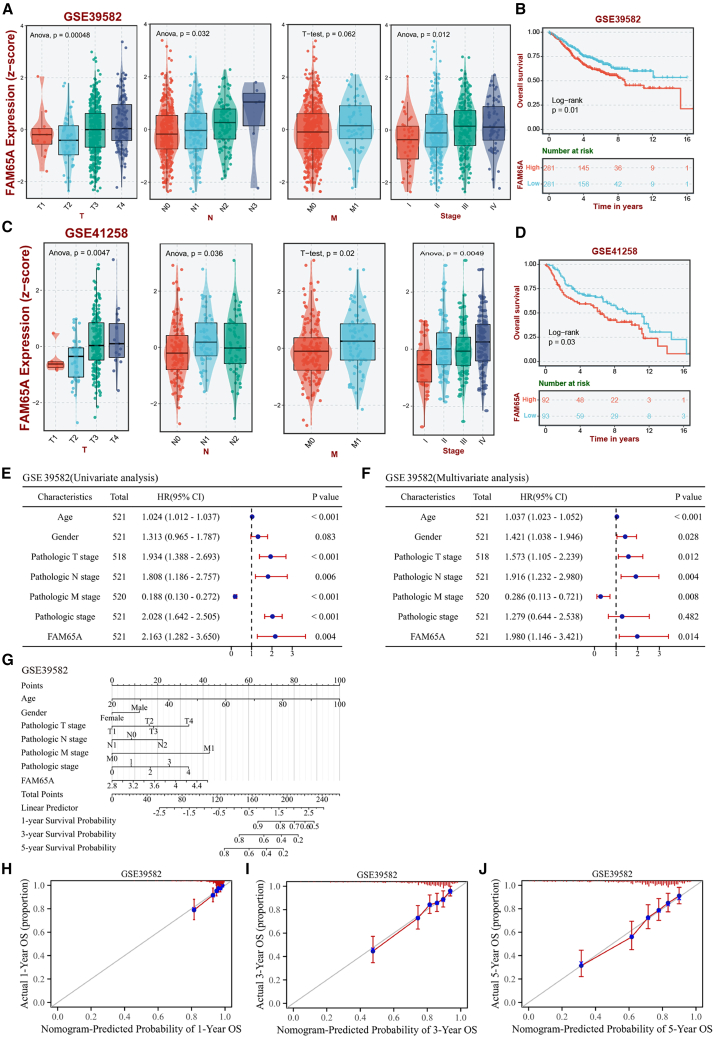
*FAM65A* as an independent prognostic biomarker correlated with clinicopathological features in CRC patients (A and C) Correlation between *FAM65A* expression and T-, N-, and M-stages in GEO: GSE39582 and GEO: GSE41258 datasets. (B and D) Prognostic relationship between *FAM65A* and OS of colon cancer patients in GEO: GSE39582 and GEO: GSE41258 datasets. (E and F) The results of both univariate and multivariate Cox regression analyses conducted on GEO: GSE39582 dataset. (G) The construction of a nomogram model incorporating *FAM65A* within GEO: GSE39582 dataset. (H–J) The calibration curves for the 1-, 3-, and 5-year nomograms. Data are presented as mean ± SEM.

We began by examining the relationship between *FAM65A* expression and various clinicopathological characteristics using data from the GEO database. Our analysis of both the GEO: GSE39582 and GEO: GSE41258 datasets indicated that colon cancer patients exhibiting higher T-stage, N-stage, and pathological stage also had elevated levels of *FAM65A* expression (Figures 2A and 2C). Additionally, patients categorized in the high *FAM65A* expression group experienced shorter overall survival (OS) rates (Figures 2B and 2D). To validate these findings, we further analyzed data from the TCGA database, which largely corroborated the results obtained from the GEO dataset (Figure S2A).

Furthermore, we observed that *FAM65A* expression was significantly elevated in tissues exhibiting lymphoid and perineural infiltration, with no notable correlation to age or gender (Figure S2B). Our investigation into the prognostic implications of *FAM65A* in colon cancer revealed that higher expression levels were significantly linked to poorer OS, disease-specific survival, and a less favorable prognosis for progression-free intervals (Figures S2C–S2E). In summary, our findings underscore the prognostic significance of *FAM65A* in relation to the clinicopathological parameters of colon cancer, indicating an association with a poorer prognosis.

### *FAM65A* was identified as an independent prognostic indicator for CRC patients

We evaluated the prognostic significance of *FAM65A* expression alongside pathological stage and T/N/M classification in colon cancer patients. Both univariate and multivariate Cox regression analyses indicated that *FAM65A* serves as an independent prognostic factor for OS in colon cancer patients, as evidenced by data from the TCGA database (Figures S2F and S2G). Furthermore, similar results were observed in the GEO: GSE39582 dataset (Figures 2E and 2F). These findings underscore the potential of *FAM65A* expression as a reliable independent prognostic marker in this patient population.

Additionally, we established that *FAM65A* is a robust predictor of 1-, 3-, and 5-year survival outcomes by developing a column-line graphical model (Figures S2H and S2G). Calibration curves further corroborated these results (Figures 2H–2J and S2I–S2K). Collectively, these experimental outcomes suggest that *FAM65A* may be utilized as an independent prognostic predictor for patients diagnosed with colon cancer.

### Knockdown of *FAM65A* expression inhibits cell proliferation *in vitro*

**Figure 3:**
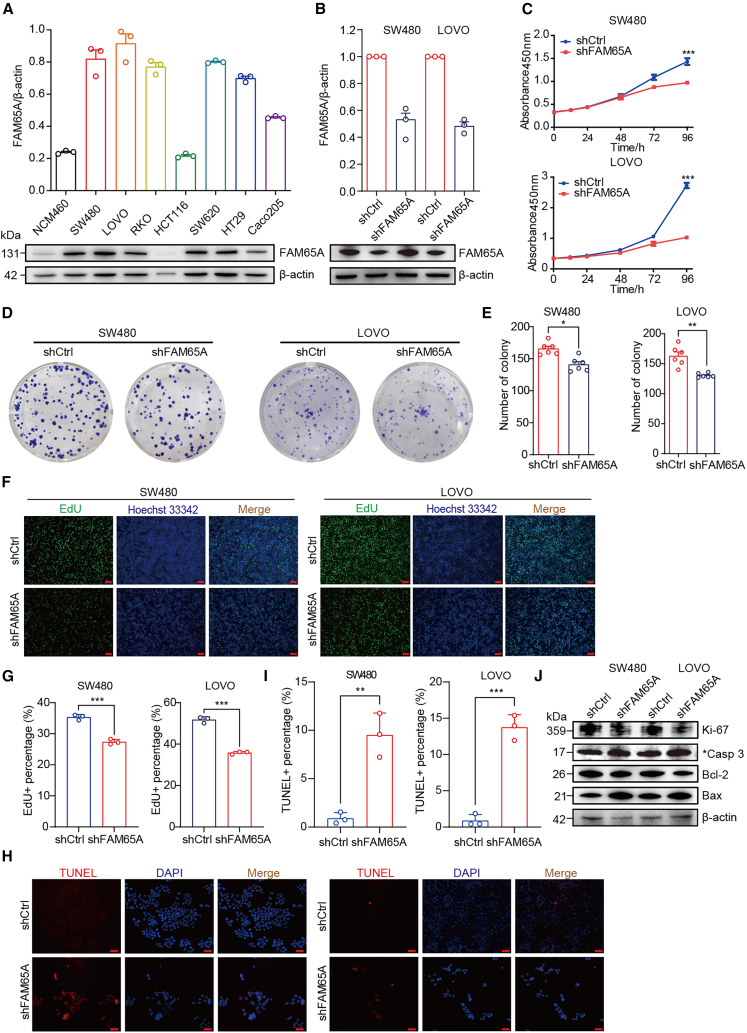
FAM65A promotes CRC cell proliferation and inhibits cell apoptosis (A) Western blot analysis FAM65A expression in human normal colon cells and CRC cells was assessed. (B) Western blot analysis FAM65A knockdown efficiency in SW480 and LOVO cells. (C) CCK8 assay was performed with shCtrl and shFAM65A in SW480 and LOVO cell lines, *n* = 3, ∗∗∗*p* < 0.001. (D) The outcomes of the colony formation assay for shCtrl and shFAM65A in SW480 and LOVO cells were reported. (E) A quantitative analysis of the colony formation assay was conducted, *n* = 6, ∗*p* < 0.05, ∗∗*p* < 0.01. (F) The results of the EdU assay for shCtrl and shFAM65A in SW480 and LOVO cells were presented, where EdU-positive stained cells exhibited green fluorescence and nuclei displayed blue fluorescence. Scale bars, 100 μm. (G) A quantitative analysis of the EdU assay results was performed, *n* = 3, ∗∗∗*p* < 0.001. (H) The results of the apoptosis assay for shCtrl and shFAM65A in SW480 and LOVO cells were documented. Scale bars, 50 μm. (I) A quantitative analysis of the apoptosis assay was conducted, *n* = 3, ∗∗*p* < 0.01, ∗∗∗*p* < 0.001. (J) The western blot results for the expression of Ki-67, cleaved caspase 3, Bcl-2, and Bax in SW480 and LOVO cells for both shCtrl and shFAM65A were presented. Data are presented as mean ± SEM of biologically independent experiments.

To verify the reproducibility of the aforementioned findings in both normal and CRC cell lines *in vitro*, we employed western blot analysis. The results depicted in Figure 3A indicate that *FAM65A* is significantly overexpressed in CRC cells compared to the human normal colonic epithelial cell line NCM460. Notably, *FAM65A* expression was lower in HCT116 cells relative to other CRC cell lines, while SW480 and LOVO cells exhibited higher levels of expression. Consequently, we proceeded to knock down FAM65A expression in SW480 and LOVO cells, confirming the knockdown via western blot (Figure 3B). To assess the impact of FAM65A on CRC cell proliferation *in vitro*, we conducted CCK8, colony formation, and EdU assays using shCtrl and shFAM65A cells. The CCK8 assay results demonstrated a reduction in the proliferative capacity of FAM65A knockdown cells in both SW480 and LOVO lines (Figure 3C). Furthermore, the colony formation assay illustrated that the knockdown of FAM65A inhibited the clonogenic potential of SW480 and LOVO cells (Figures 3D and 3E). EdU assays further corroborated these findings, revealing a decrease in the rate of EdU^+^ cells and DNA replication following FAM65A knockdown (Figures 3F and 3G). Additionally, western blot analysis was utilized to evaluate the expression of molecular markers associated with proliferation, revealing that FAM65A knockdown led to a downregulation of Ki-67 (Figure 3J). Collectively, these results suggest that the downregulation of FAM65A impedes CRC cell proliferation.

### Knockdown of *FAM65A* expression promotes cell apoptosis *in vitro*

Subsequently, we conducted apoptosis assays to further investigate the impact of FAM65A knockdown on apoptosis in CRC cells. The results illustrated in Figures 3H and 3I demonstrate that the apoptotic ratio in shFAM65A cells was significantly higher than that in shCtrl cells across both SW480 and LOVO cell lines. Furthermore, western blot analysis revealed that the knockdown of FAM65A resulted in a downregulation of the anti-apoptotic protein Bcl-2, while simultaneously enhancing the expression of pro-apoptotic proteins cleaved Caspase 3 and Bax (Figure 3J). Therefore, it can be concluded that *FAM65A* plays a role in promoting CRC cell proliferation and inhibiting apoptosis *in vitro*.

### Knockdown of FAM65A expression represses cell migration *in vitro*

**Figure 4:**
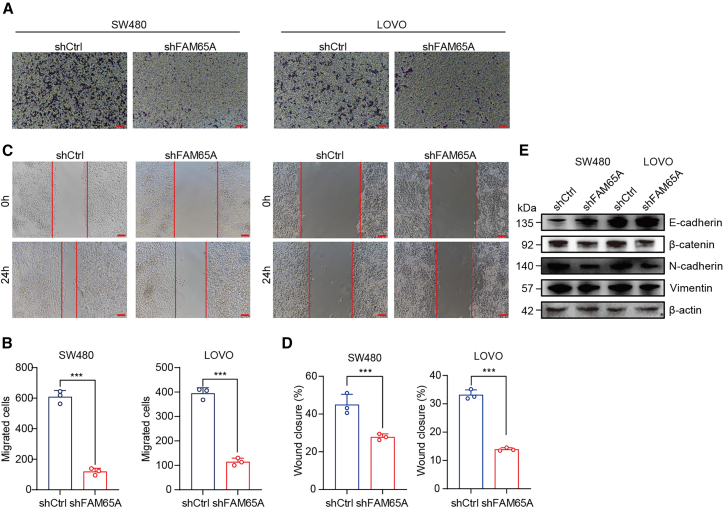
FAM65A promotes CRC cell migration *in vitro* (A) *Transwell* migration assay was conducted to evaluate the migration capabilities of shCtrl and shFAM65A in SW480 and LOVO cell lines. Scale bars, 50 μm. (B) A quantitative analysis of the *Transwell* migration assay was performed, *n* = 3, ∗∗∗*p* < 0.001. (C) The results of wound healing assay comparing shCtrl and shFAM65A in SW480 and LOVO cells were obtained. Scale bars, 50 μm. (D) A quantitative analysis of the wound healing assay was also conducted, *n* = 3, ∗∗∗*p* < 0.001. (E) Western blot analysis was performed to assess the expression levels of EMT markers in SW480 and LOVO cells. Data are presented as mean ± SEM of biologically independent experiments.

To investigate the role of FAM65A in CRC cell migration, we conducted *transwell* migration assays in SW480 and LOVO cell lines. The results depicted in Figures 4A and 4B demonstrate that FAM65A knockdown resulted in a diminished migratory capacity of the cells. Consistent with these findings, wound healing assays indicated a reduced percentage of wound closure following FAM65A knockdown (Figures 4C and 4D). Furthermore, western blot analysis of EMT (epithelial-mesenchymal transition)-related markers revealed that *FAM65A* knockdown decreased the expression of β-catenin, N-cadherin, and vimentin, while increasing E-cadherin expression (Figure 4E).

### Ectopic expression of *FAM65A* facilitates cell proliferation and migration and suppresses cell apoptosis *in vitro*

**Figure 5:**
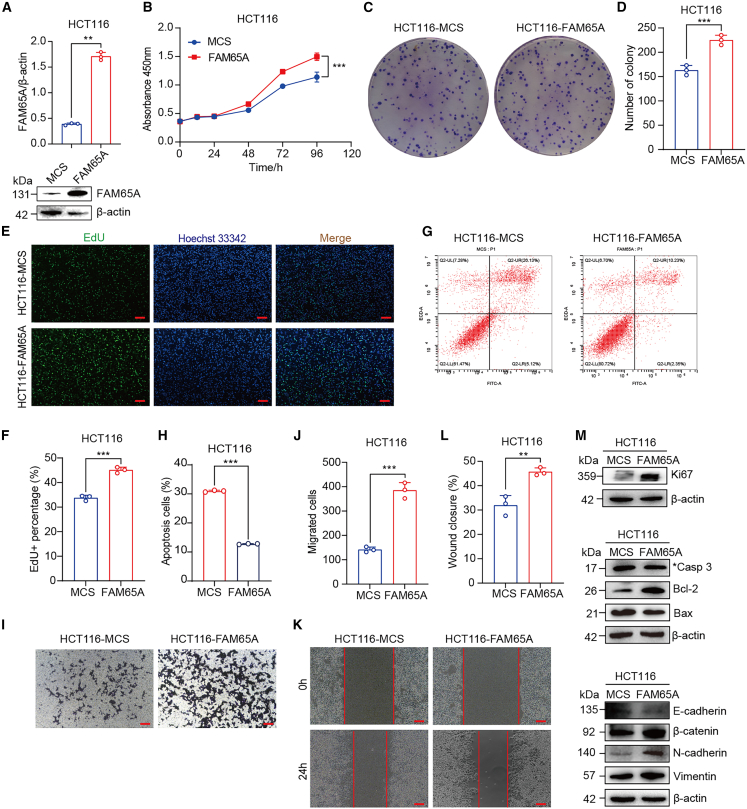
Ectopic expression of FAM65A promotes CRC cell proliferation, migration, and inhibits cell apoptosis *in vitro* (A) Western blot analysis the efficiency of FAM65A overexpression, accompanied by a quantitative assessment, *n* = 3, ∗∗*p* < 0.01. (B) The findings from the CCK8 assay conducted on HCT116-MCS and HCT116-FAM65A cells are presented, *n* = 3, ∗∗∗*p* < 0.001. (C) The outcomes of the colony formation assay for HCT116-MCS and HCT116-FAM65A cells are reported. (D) A quantitative analysis of the colony formation assay results is provided, *n* = 3, ∗∗∗*p* < 0.001. (E) The results of the EdU assay in HCT116-MCS and HCT116-FAM65A cells are shown. Scale bars, 100 μm. (F) A quantitative analysis of the EdU assay results is included, *n* = 3, ∗∗∗*p* < 0.001. (G) The findings from the apoptosis assay in HCT116-MCS and HCT116-FAM65A cells are presented. (H) A quantitative analysis of the apoptosis assay results is provided, *n* = 3, ∗∗∗*p* < 0.001. (I) The results of the *transwell* migration assay for HCT116-MCS and HCT116-FAM65A cells are reported. Scale bars, 50 μm. (J) A quantitative analysis of the *transwell* migration assay results is included, *n* = 3, ∗∗∗*p* < 0.001. (K) The outcomes of the wound healing assay in HCT116-MCS and HCT116-FAM65A cells are presented. Scale bars, 50 μm. (L) A quantitative analysis of the wound healing assay results is provided, *n* = 3, ∗∗*p* < 0.01. (M) The western blot analysis illustrates the expression levels of Ki-67, cleaved caspase 3, Bcl-2, Bax, E-cadherin, N-cadherin, β-catenin, and vimentin in HCT116-MCS and HCT116-FAM65A cells. Data are presented as mean ± SEM of biologically independent experiments.

To further elucidate the functional role of FAM65A in CRC cells, we ectopically expressed FAM65A in the HCT116 cell line. Western blot analysis confirmed the successful overexpression of FAM65A in HCT116 cells (Figure 5A). To assess the impact of FAM65A overexpression on CRC cell proliferation, we employed CCK8 assays, which indicated that FAM65A overexpression significantly enhanced cell growth rates at specific time points (Figure 5B). Colony formation assays corroborated these findings, revealing an increase in the number of cell colonies formed following FAM65A overexpression (Figures 5C and 5D). Additionally, EdU assays demonstrated that FAM65A overexpression augmented the proliferative capacity of HCT116 cells (Figures 5E and 5F). These results collectively indicate that FAM65A overexpression promotes CRC cell proliferation.

Subsequently, we conducted apoptosis assays to evaluate the effect of FAM65A on cell apoptosis, revealing that increased FAM65A expression inhibited apoptosis in HCT116 cells (Figures 5G and 5H). Moreover, we investigated the role of FAM65A overexpression in CRC cell migration. *Transwell* migration assays indicated that FAM65A overexpression enhanced the migratory ability of HCT116 cells (Figures 5I and 5J). Similar results were observed in wound healing assays, where FAM65A overexpression facilitated HCT116 cell migration (Figures 5K and 5L). Western blot analysis further revealed that FAM65A overexpression increased the expression of Ki-67, Bcl-2, and mesenchymal markers (β-catenin, N-cadherin, and vimentin), while decreasing the expression of cleaved caspase 3, Bax, and epithelial markers (E-cadherin) (Figure 5M). These findings suggest that FAM65A overexpression promotes CRC cell proliferation, migration, and inhibits apoptosis *in vitro*.

### FAM65A mediates RSK activation to promote CRC progression

**Figure 6:**
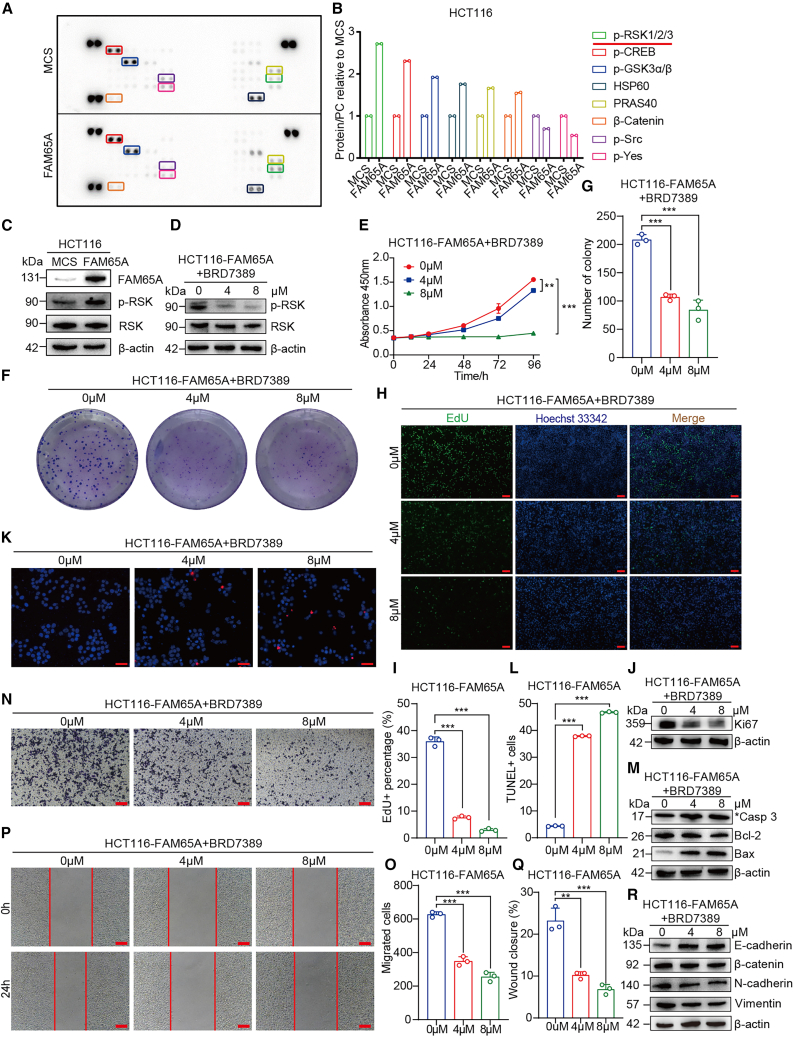
FAM65A promotes CRC tumor progression is dependent on RSK phosphorylation (A) Results from protein kinase microarray analysis of HCT116-MCS and HCT116-FAM65A cells. (B) Quantitative assessment of the protein kinase microarray data, *n* = 2. (C) Western blot analysis demonstrating the expression levels of *p*-RSK and total RSK in HCT116-MCS and HCT116-FAM65A cells. (D) Western blot analysis of *p*-RSK and RSK expression in HCT116-FAM65A cells treated with 4 and 8 μM BRD7389 or without treatment. (E) Results from the CCK8 cell proliferation assay conducted on HCT116-FAM65A cells with and without the application of BRD7389, *n* = 3, ∗∗*p* < 0.01, ∗∗∗*p* < 0.001. (F) Outcomes of the colony formation assay performed on HCT116-FAM65A cells treated with BRD7389 or without treatment. (G) Quantitative analysis of the colony formation assay results, *n* = 3, ∗∗∗*p* < 0.001. (H) Results from the EdU assay conducted on HCT116-FAM65A cells with and without the application of BRD7389. Scale bars, 100 μm. (I) Quantitative analysis of the EdU assay results, *n* = 3, ∗∗∗*p* < 0.001. (J) Western blot analysis of Ki-67 expression in HCT116-FAM65A cells treated with BRD7389 or not. (K) Results from the apoptosis assay conducted on HCT116-FAM65A cells treated with BRD7389 or not. Scale bars, 50 μm. (L) Quantitative analysis of the apoptosis experiments, *n* = 3, ∗∗∗*p* < 0.001. (M) Western blot analysis of the expression levels of cleaved Caspase 3, Bcl-2, and Bax in HCT116-FAM65A cells treated with BRD7389 or not. (N) Results from the *Transwell* migration assay conducted on HCT116-FAM65A cells with and without the application of BRD7389. Scale bars, 50 μm. (O) Quantitative analysis of the *Transwell* migration assay results, *n* = 3, ∗∗∗*p* < 0.001. (P) Results from the wound healing assay performed on HCT116-FAM65A cells treated with BRD7389 or not. Scale bars, 50 μm. (Q) Quantitative analysis of the wound healing assay results, *n* = 3, ∗∗*p* < 0.01, ∗∗∗*p* < 0.001. (R) Western blot analysis the expression of EMT markers in HCT116-FAM65A cells treated with BRD7389 or not. Data are presented as mean ± SEM of biologically independent experiments.

To elucidate the molecular mechanisms underlying FAM65A-mediated CRC tumor progression, we performed a protein kinase microarray analysis in HCT116-MCS and HCT116-FAM65A cells, assessing the phosphorylation levels of 37 kinases. The results presented in Figures 6A and 6B indicate that FAM65A overexpression led to an increase in the expression of various phosphokinases, particularly *p*-RSK. Given the established role of *p*-RSK in regulating CRC cell proliferation, migration, and apoptosis, we focused our investigation on *p*-RSK. Western blot analysis confirmed that FAM65A overexpression resulted in elevated levels of *p*-RSK in HCT116 cells (Figure 6C). To assess the functional significance of RSK phosphorylation in FAM65A-mediated CRC cell behavior, we inhibited RSK phosphorylation using the RSK family kinase inhibitor BRD7389. As demonstrated in Figure 6D, both 4 and 8 μM concentrations of BRD7389 effectively reduced RSK phosphorylation in HCT116-FAM65A cells.

We subsequently explored the effects of BRD7389 on proliferation, migration, and apoptosis in HCT116-FAM65A cells. CCK8 assays revealed that the RSK inhibitor BRD7389 was capable of reversing the enhanced growth rate induced by FAM65A overexpression at specific time points (Figure 6E). Colony formation assays further demonstrated that BRD7389 inhibited the number of colonies formed in response to FAM65A overexpression (Figures 6F and 6G). Additionally, EdU assays indicated that the proliferation enhancement conferred by FAM65A expression was inhibited by the RSK inhibitor (Figures 6H and 6I). Western blot analysis confirmed that BRD7389 reduced the expression of Ki-67 that were elevated by FAM65A overexpression (Figure 6J). Furthermore, apoptosis assays indicated that the RSK inhibitor BRD7389 promoted apoptosis in HCT116-FAM65A cells, as evidenced by increased levels of cleaved Caspase 3 and Bax, and decreased Bcl-2 expression (Figures 6K and 6L).

We also examined the impact of the RSK inhibitor BRD7389 on the migratory capacity of HCT116-FAM65A cells. *Transwell* migration assays demonstrated that the migratory ability enhanced by high levels of FAM65A expression was inhibited by the RSK inhibitor (Figures 6N and 6O). Similar results were observed in wound healing assays, where reduced RSK phosphorylation levels inhibited the migratory capacity of HCT116 cells promoted by FAM65A overexpression (Figures 6P and 6Q). Additionally, Western blot analysis revealed that the RSK inhibitor increased E-cadherin expression while decreasing the expression of mesenchymal markers such as β-catenin, N-cadherin, and vimentin (Figure 6R). These findings suggest that inhibiting RSK phosphorylation effectively reverses the effects of FAM65A overexpression on CRC cell proliferation, migration, and apoptosis *in vitro*.

### FAM65A binds to Ras and activates the Ras/ERK signaling to mediate RSK activation

**Figure 7:**
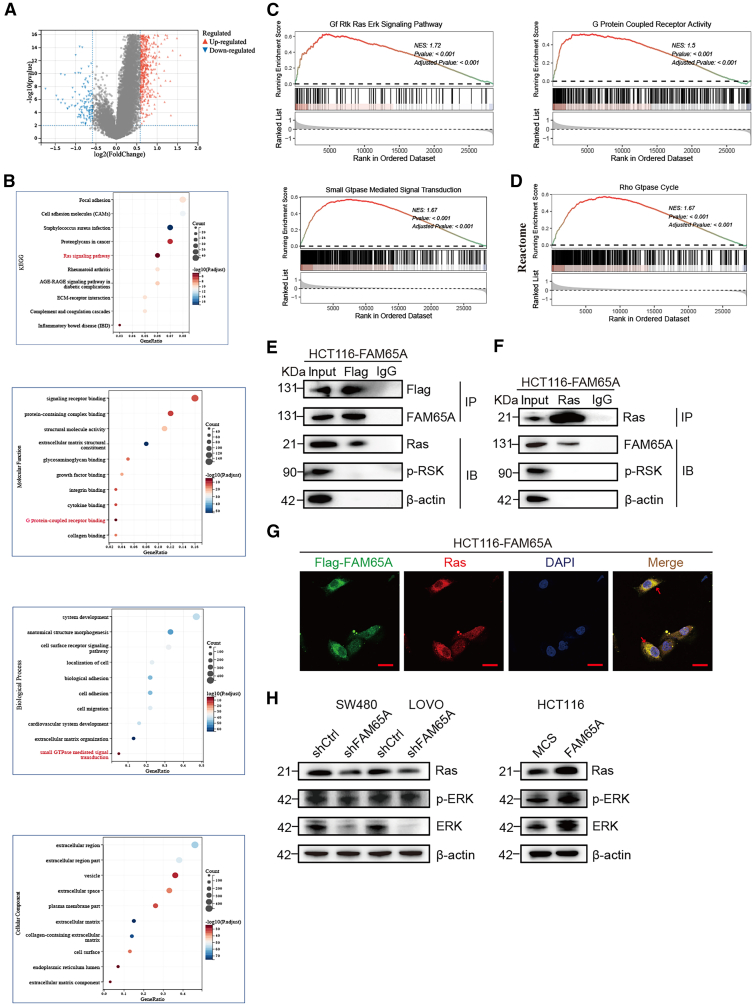
FAM65A binds to Ras and activates the Ras/ERK signaling to mediate RSK activation (A) The volcano plot analysis results for the *FAM65A* high-expression and low-expression groups from the TCGA database were shown. (B) The KEGG and GO results were shown. (C) The GSEA results were shown. (D) GSEA on DEGs between the *FAM65A* high-expression group and low-expression group in the Reactome database were shown. (E) IP was performed to detect the binding of FAM65A and Ras/p-RSK. (F) IP was performed to detect the binding of Ras and FAM65A/p-RSK. (G) Immunofluorescence was performed to detect the co-localization of FAM65A and Ras. Scale bars, 20 μm. (H) Western blot analysis the Ras and *p*-ERK expression in FAM65A knockdown or overexpression cells. Data are presented as mean ± SEM of biologically independent experiments.

To elucidate the molecular mechanism through which FAM65A activates RSK, RNA sequencing data from the TCGA database were analyzed. Samples were stratified into low- and high-expression groups based on the median expression level of *FAM65A*. Differential gene expression analysis identified 996 genes upregulated and 114 genes downregulated in the high-expression group relative to the low-expression group (Figure 7A). Subsequent Kyoto Encyclopedia of Genes and Genomes (KEGG) and Gene Set Enrichment Analysis (GSEA) results indicated that the differentially expressed genes were significantly enriched in the Ras signaling pathway, with elevated *FAM65A* expression correlating with activation of this pathway (Figures 7B and 7C). Additionally, Gene Ontology (GO) enrichment analysis revealed that FAM65A exhibits molecular functions related to G protein-coupled receptor activity and is involved in small GTPase-mediated signal transduction processes (Figure 7B). GSEA conducted using the Reactome database further demonstrated upregulation of the Rho GTPase cycle pathway in the *FAM65A*-high group compared to the low-expression group (Figure 7D). These data suggest that *FAM65A* participates in small GTPase-mediated signal transduction and facilitates colon cancer progression through modulation of the Ras signaling pathway.

To validate these observations, immunoprecipitation assays were performed in HCT116 cells overexpressing FAM65A. Whether performing pull-down using flag-FAM65A to detect *p*-RSK and Ras (Figure 7E), or using Ras antibody for pull-down to detect FAM65A and Ras (Figure 7F), the results consistently demonstrate that FAM65A binds to Ras, while neither FAM65A nor Ras exhibits binding to *p*-RSK. Immunofluorescence analyses further corroborated these findings by showing pronounced colocalization of FAM65A and Ras within the cytoplasm (Figure 7G).

Subsequently, FAM65A knockdown or overexpression cells were employed to assess the expression levels of Ras and *p*-ERK. The results showed that FAM65A knockdown decreased expression of both Ras and *p*-ERK, whereas FAM65A overexpression increased levels of these proteins (Figure 7H).

Taken together, these results indicate that FAM65A facilitates RSK activation by interacting with Ras and promoting activation of the Ras/ERK signaling cascade.

### Ras/ERK signaling activation was indispensable for FAM65A-mediated RSK activation and CRC progression

**Figure 8:**
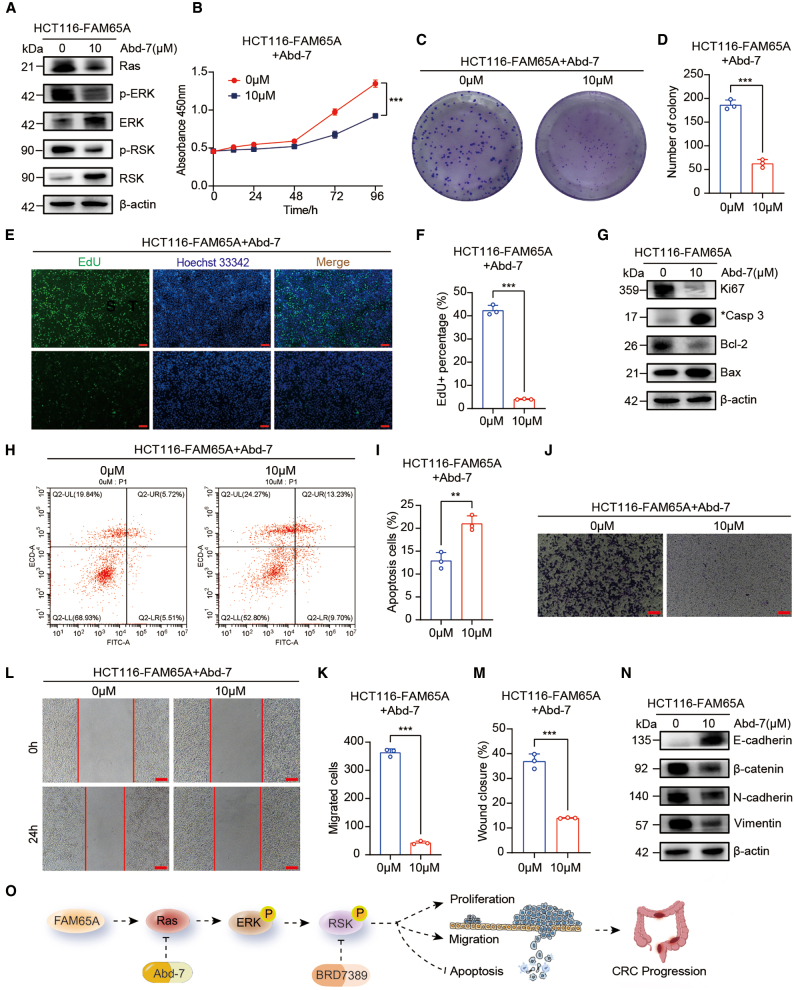
Ras/ERK signaling activation was indispensable for FAM65A-mediated RSK activation and CRC progression (A) Western blot analysis of Ras and *p*-ERK expression in HCT116-FAM65A cells treated with 10 μM Abd-7, or without treatment. (B) Results from the CCK8 cell proliferation assay conducted on HCT116-FAM65A cells with and without the application of Abd-7, *n* = 3, ∗∗∗*p* < 0.001. (C) Colony formation assay performed on HCT116-FAM65A cells treated with Abd-7 or not. (D) Quantitative analysis of the colony formation assay results, *n* = 3, ∗∗∗*p* < 0.001. (E) Results from the EdU assay conducted on HCT116-FAM65A cells with and without the application of Abd-7. Scale bars, 100 μm. (F) Quantitative analysis of the EdU assay results, *n* = 3, ∗∗∗*p* < 0.001. (G) Western blot analysis of Ki-67, cleaved Caspase 3, Bcl-2, and Bax expression in HCT116-FAM65A cells treated with Abd-7 or not. (H) Results from the apoptosis assay conducted on HCT116-FAM65A cells treated with Abd-7 or not. Scale bars, 50 μm. (I) Quantitative analysis of the apoptosis experiments, *n* = 3, ∗∗∗*p* < 0.001. (J) Results from the *Transwell* migration assay conducted on HCT116-FAM65A cells with and without the application of Abd-7. Scale bars, 50 μm. (K) Quantitative analysis of the *Transwell* migration assay results, *n* = 3, ∗∗∗*p* < 0.001. (L) Results from the wound healing assay performed on HCT116-FAM65A cells treated with Abd-7 or not. Scale bars, 50 μm. (M) Quantitative analysis of the wound healing assay results, *n* = 3, ∗∗∗*p* < 0.001. (N) Western blot analysis the expression of EMT markers in HCT116-FAM65A cells treated with Abd-7 or not. (O) Proposed model of FAM65A in CRC progression. Data are presented as mean ± SEM of biologically independent experiments.

To substantiate the involvement of Ras activation in FAM65A-mediated RSK activation and the malignant progression of CRC, we utilized the Ras-specific small-molecule inhibitor Abd-7 to suppress Ras signaling and its downstream effectors. Western blot analysis demonstrated that treatment with 10 μM Abd-7 effectively inhibited the activation of Ras and its downstream target ERK, concomitantly attenuating RSK activation (Figure 8A). Subsequently, we assessed the impact of Abd-7 on cellular proliferation, migration, and apoptosis in HCT116 cells overexpressing FAM65A. CCK8 assays indicated that Abd-7 reversed the proliferation enhancement induced by FAM65A overexpression at designated time points (Figure 8B). Colony formation assays corroborated these results, showing a reduction in colony numbers following Abd-7 treatment in FAM65A-overexpressing cells (Figures 8C and 8D). Furthermore, EdU incorporation assays revealed that Abd-7 inhibited the proliferative advantage conferred by FAM65A expression (Figures 8E and 8F). Western blot analysis confirmed that Abd-7 decreased the expression of the proliferation marker Ki-67 induced by FAM65A overexpression (Figure 8G). Apoptosis assays demonstrated that Abd-7 promoted apoptotic processes in HCT116-FAM65A cells, as evidenced by increased levels of cleaved Caspase 3 and Bax, alongside a reduction in Bcl-2 expression (Figures 8G–8I).

We further investigated the influence of Abd-7 on the migratory capacity of HCT116-FAM65A cells. *Transwell* migration assays revealed that Abd-7 suppressed the enhanced migratory ability associated with elevated FAM65A expression (Figures 8J and 8K). Consistent results were obtained from wound-healing assays, wherein Abd-7 inhibited FAM65A overexpression-induced migration in HCT116 cells (Figures 8L and 8M). Western blot analysis further demonstrated that Abd-7 treatment upregulated the epithelial marker E-cadherin while downregulating mesenchymal markers, including β-catenin, N-cadherin, and vimentin (Figure 8N).

Collectively, these findings indicate that inhibition of Ras pathway activation effectively counteracts the effects of FAM65A overexpression on RSK activation and modulates CRC cell proliferation, migration, and apoptosis *in vitro* (Figure 8O).

### Knockdown of FAM65A inhibits tumor progression *in vivo*

**Figure 9:**
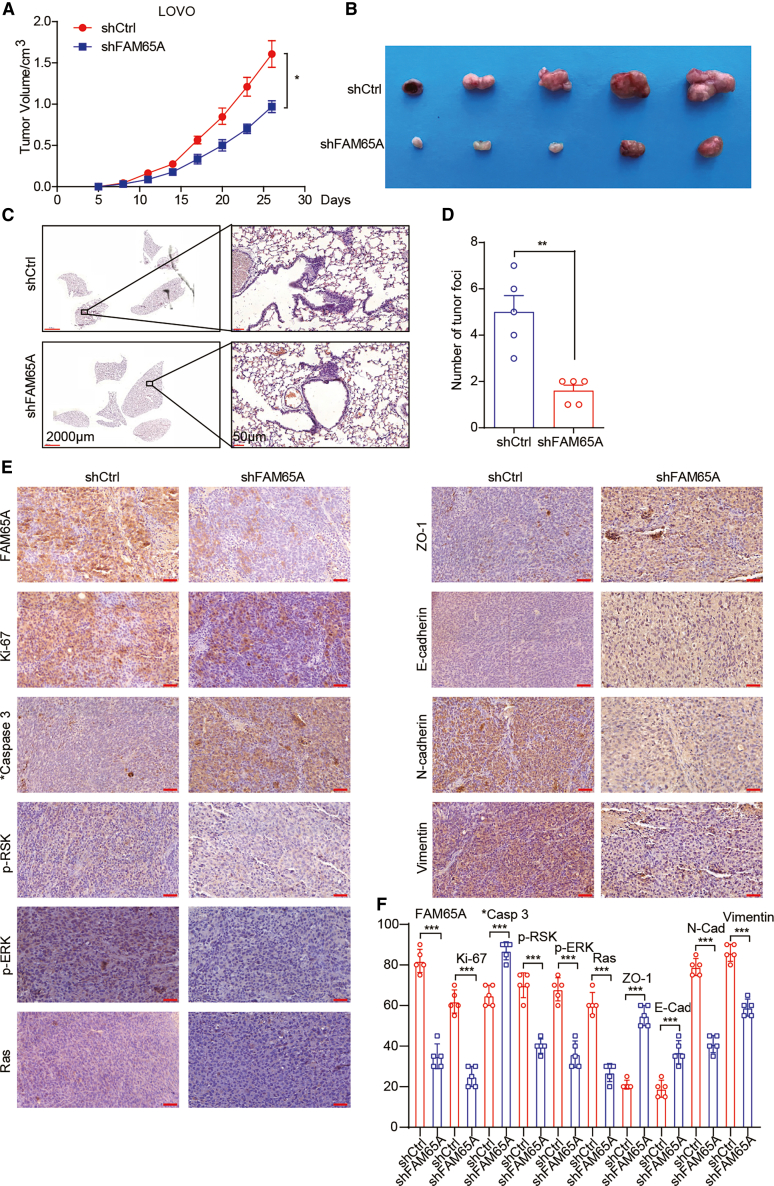
Knockdown of FAM65A inhibits tumor progression *in vivo* (A) LOVO-shCtrl and LOVO-shFAM65A cells were administered into the fourth fat pad of nude mice, and the resulting tumor growth curves were subsequently generated, *n* = 5, ∗*p* < 0.05. (B) The tumors excised from mice across various experimental groups are presented. (C) Hematoxylin and Eosin (HE) staining results of lung tissue from the different groups is displayed. (D) A quantitative analysis of metastatic lung nodules is provided, *n* = 5, ∗∗*p* < 0.01. (E) IHC results for FAM65A, Ki-67, *p*-RSK, *p*-ERK, Ras, N-cadherin, vimentin, cleaved Caspase 3, ZO-1, and E-cadherin in tumor tissues are illustrated. (F) A quantitative analysis of the IHC results is included. Data are presented as mean ± SEM of biologically independent experiments, *n* = 5, ∗∗∗*p* < 0.001.

Finally, we investigated the role of FAM65A in tumor progression using a xenograft mouse model. We injected LOVO-shCtrl and LOVO-shFAM65A cells into the fourth fat pad of nude mice. The results indicated a significant reduction in tumor growth rate and volume in the LOVO-shFAM65A group compared to the control group (Figures 9A and 9B). Furthermore, histological examination of mouse lung tissues revealed a marked decrease in the number of metastatic nodules in the LOVO-shFAM65A group (Figures 9C and 9D). Immunohistochemical staining of tumor tissues (Figures 9E and 9F) demonstrated that FAM65A knockdown resulted in decreased expression of Ki-67, *p*-RSK, *p*-ERK, Ras, N-cadherin, and vimentin, while increasing the expression of cleaved Caspase 3, ZO-1, and E-cadherin, consistent with our *in vitro* findings. In conclusion, these results suggest that the downregulation of FAM65A expression inhibits cancer progression *in vivo*.

### Proposed model of FAM65A in CRC clinicopathological features, prognosis, and progression

**Figure 10:**
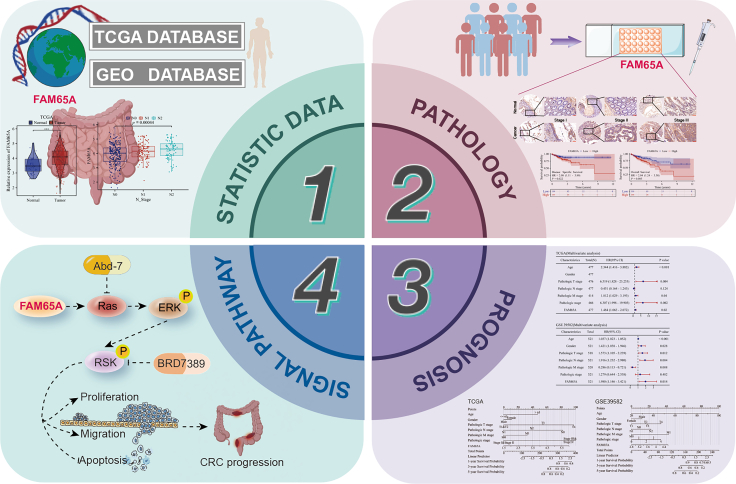
Proposed model of FAM65A in CRC clinicopathological features, prognosis, and progression

Based on these findings, we propose the following model (Figure 10): *FAM65A* functions as a potential oncogene, exhibiting elevated expression levels in CRC and demonstrating a correlation with various clinicopathological characteristics and patient prognosis. Notably, *FAM65A* serves as an independent prognostic indicator for individuals diagnosed with CRC. Investigations into the underlying mechanisms revealed that FAM65A binds to Ras and activates the Ras/ERK signaling to mediate RSK activation contributes to the malignant progression of CRC.

## Discussion

CRC ranks among the most prevalent malignant solid tumors globally, characterized by elevated incidence and mortality rates.^1^ Despite the continuous advancement and refinement of clinical treatment modalities, the late-stage diagnosis and subsequent metastasis of the disease pose considerable challenges in its management.^22^ The ongoing evolution of precision medicine offers the potential for identifying early diagnostic markers and therapeutic targets,^23^ which may yield innovative strategies for the treatment of CRC.

Recent investigations have identified *FAM65A* as a prognostic biomarker for human malignancies through pan-cancer analyses.^12^ In this study, analysis of publicly available datasets from the TCGA and GEO repositories demonstrated that overexpression of *FAM65A* is significantly correlated with OS, disease-free survival (DFS), and pathological staging in patients with CRC. Nevertheless, given the limited sample size, the relationship between *FAM65A* expression and the pathological features and prognosis of CRC warrants validation in larger clinical cohorts. Moreover, the potential role of *FAM65A* as an early diagnostic biomarker remains to be elucidated, highlighting the need for further investigation into its underlying molecular mechanisms and clinical applicability. Future research should prioritize increasing sample sizes to confirm the prognostic relevance of *FAM65A* expression in CRC. Subsequently, comprehensive studies exploring the molecular pathways involving *FAM65A* in tumorigenesis and disease progression are essential. Finally, rigorous assessment of the sensitivity and specificity of *FAM65A* as an early diagnostic marker is necessary to substantiate its potential for clinical implementation.

The molecular mechanisms underlying the role of FAM65A in tumorigenesis have yet to be fully elucidated. A principal contribution of the present study is the demonstration that FAM65A facilitates malignant progression in CRC by interacting with Ras and subsequently activating the Ras/ERK signaling cascade, which in turn mediates RSK activation. This finding offers a framework for understanding the mechanistic functions of FAM65A. Nonetheless, several intermediate aspects remain to be clarified, including whether FAM65A associates with Ras proteins through direct or indirect interactions, and whether this binding is modulated by upstream signaling events. Additionally, the regulatory mechanisms responsible for the elevated expression of FAM65A in CRC, particularly the upstream signaling pathways involved, have not been characterized. Future investigations should aim to delineate the upstream regulatory networks controlling FAM65A expression, as well as the precise molecular processes by which FAM65A binds to and activates Ras.

The RSK family, classified as serine/threonine protein kinases, has been shown to regulate the stability of eukaryotic translation initiation factors (eIFs),^24^ thereby modulating protein synthesis. As a well-established downstream kinase target of the Ras/ERK or MAPK signaling pathways,^25^^,^^26^ prior research has demonstrated a correlation between RSK activation and both colonic inflammation and CRC progression.^27^^,^^28^^,^^29^ In contrast, the present study elucidates a functional mechanism involving FAM65A, wherein FAM65A interacts with Ras to activate Ras/ERK signaling, subsequently promoting RSK activation. This finding suggests a potential therapeutic target for CRC. Furthermore, inhibition of RSK activation significantly impeded FAM65A-driven CRC progression, underscoring the therapeutic potential of targeting this pathway in CRC treatment.

In summary, our research identifies FAM65A as a potential oncogene with increased expression in CRC, correlating with various clinicopathological features and patient outcomes. It highlights FAM65A as an independent prognostic marker for CRC. Experimental results show that FAM65A facilitates CRC cell proliferation and metastasis cell proliferation and migration while suppressing apoptosis both *in vitro* and *in vivo*. Investigations into the underlying mechanisms revealed that FAM65A binds to Ras and activates the Ras/ERK signaling to mediate RSK activation, contributing to the malignant progression of CRC; treatment with the Ras inhibitor Abd-7 or RSK inhibitor BRD7389 effectively countered the FAM65A-mediated enhancement of malignancy. Overall, the findings suggest that FAM65A could serve as a valuable biomarker and therapeutic target for CRC.

### Limitations of the study

This study conducted an analysis of publicly accessible datasets obtained from the TCGA and GEO repositories, revealing a significant association between *FAM65A* expression, OS, DFS, as well as pathological staging in patients with CRC. Nonetheless, due to the limited sample size, the relationship between *FAM65A* expression and the pathological features and prognosis of CRC warrants validation in larger clinical cohorts. Additionally, the potential utility of *FAM65A* as an early diagnostic biomarker remains to be fully elucidated. Future investigations should focus on increasing sample sizes to substantiate the prognostic significance of *FAM65A* expression in CRC. Moreover, while the present study establishes that FAM65A facilitates malignant progression in CRC through interaction with the Ras protein—thereby activating the Ras/ERK signaling pathway and mediating RSK activation—several mechanistic details remain unclear. Specifically, it is necessary to determine whether the interaction between FAM65A and Ras is direct or indirect, and to elucidate whether this interaction is modulated by upstream signaling events.

## Resource availability

### Lead contact

Further information and requests for resources and reagents should be directed to and will be fulfilled by the lead contact, Yongming Huang (huangym0524@163.com).

### Materials availability

This study did not generate new unique reagents.

### Data and code availability

• All data reported in this article will be shared by the lead contact upon request.
• This article does not report original code. The TCGA and GEO data codes are seen in key resources table.
• Any additional information required to reanalyze the data reported in this article is available from the lead contact upon request.

## STAR★Methods

### Key resources table

**Table:** 

REAGENT or RESOURCE	SOURCE	IDENTIFIER
**Antibodies**		

β-actin	Santa Cruz Biotechnology	Cat# sc-47778; RRID: AB_626632
Epithelial-Mesenchymal Transition (EMT) Antibody Sampler Kit	Cell Signal Technology	Cat# 9782; RRID: AB_10828222
*p*-RSK	Cell Signal Technology	Cat# 11989; RRID: AB_2687613
RSK	Cell Signal Technology	Cat# 9355; RRID: AB_659900
Bcl-2	Proteintech	Cat# 12789-1-AP; RRID: AB_2227948
Ki67	Abcam	Cat# ab16667; RRID: AB_302459
Bax	Proteintech	Cat# 60267-1-Ig; RRID: AB_2848213
Ras	Proteintech	Cat# 60309-1-Ig; RRID: AB_2881422
ERK	Proteintech	Cat# 66192-1-Ig; RRID: AB_2811205
*p*-ERK	Proteintech	Cat# 28733-1-AP; RRID: AB_2881202
FAM65A	OriGene Technologies	Cat# TA335468; RRID: AB_3722673
CoraLite488-conjugated Goat Anti-Mouse IgG(H + L)	Proteintech	Cat# SA00013-1; RRID: AB_2810983
CoraLite594-conjugated Goat Anti-Rabbit IgG(H + L)	Proteintech	Cat# SA00013-4; RRID: AB_2810984

**Biological samples**		

CRC tissue microarray	Avilabio	cat. #D150Co01

**Chemicals, peptides, and recombinant proteins**		

RIPA buffer	Thermo Scientific	# 89901
ECL Protein Detection Kit	Vazyme	#E423-02
DMEM medium	Gibco	#C11995500
Fetal Bovine Serum	Gibco	# A5256701
L-glutamine	Gibco	# 25030081
sodium pyruvate	Gibco	# 11360070
NaHCO_3_	Gibco	# 25080094
NEAA	Gibco	# 11140050
McCoy’s 5A	Gibco	# 12330031
CCK-8	Dojindo	# CK04
RSK family kinase inhibitor BRD7389	MedChemExpress	# HY-12185
Protein A/G PLUS-agarose	Santa Cruz	# A3124
Ras inhibitor Abd-7	MedChemExpress	HY-122862
Avertin	Sigma-Aldrich, Shanghai	T48402

**Critical commercial assays**		

TUNEL assay	Beyotime Biotechnology	#C1089
EdU-488 assay	Beyotime Biotechnology	#C0071S
Human Phosphokinase Array Kit	R&D Systems	# ARY003C

**Deposited data**		

Expression Data from Normal and Colorectal Cancer Tissues	GEO database	GSE39582, GSE44076, GSE44861, GSE41258
RNA sequencing data from 521 colorectal cancer cases	TCGA	https://cancergenome.nih.gov/

**Experimental models: Cell lines**		

SW480 cell line	ATCC	CCL-228
LOVO cell line	ATCC	CCL-229
HCT116 cell line	ATCC	CCL-247

**Experimental models: Organisms/strains**		

BALB/c nude mice	Beijing HFK Bioscience Co. Ltd.	SPF-grade animal facility housing

**Oligonucleotides**		

FAM65A-shRNA: 5′-AAAAGCAAGTCAAGTCCATT GAATTGGATCCAATTCAATGGACTTGACTTGC-3′	This paper	–
shCtrl: 5′-AAAAGCTACACTATCGAGCAATTT TGGATCCAAAATTGCTCGATAGTGTAGC-3′	This paper	–

**Recombinant DNA**		

pLV-shRNA-puro vector	Biosettia	#B19
pLVML-3×FLAG-FAM65A	This paper	–

**Software and algorithms**		

GraphPad Prism10	GraphPad Software	https://www.graphpad.com
ImageJ	ImageJ Software	https://imagej.nih.gov/
RStudio	RStudio Software	https://posit.co/download/rstudio-desktop/
R package limma	R package limma	version 3.40.6
DAVID bioinformatics resource	DAVID bioinformatics resource	https://david.ncifcrf.gov/
Photoshop CS6	Photoshop Software	https://www.adobe.com

### Experimental model and study participant details

#### Tumor sample tissues

We used CRC tissues from patients who underwent surgery at the Affiliated Hospital of Jining Medical University from 2015 to 2019; detailed information is shown in Table S1. The CRC tissue study was approved by Jining Medical University Medical Ethics Committee (Number: JNMC-2023-YX-008). This study was also obtained informed consent from the patients, the informed consent forms are numbered as ID number.

#### Cell lines

SW480 cancer cells were cultured in DMEM (Cat. #C11995500, Gibco) supplemented with 10% FBS (Fetal Bovine Serum) (Cat. # A5256701, Gibco) and 2% L-glutamine (Cat. # 25030081, Gibco). LOVO cells were cultured in DMEM supplemented with 10% FBS, 1% sodium pyruvate (Cat. # 11360070, Gibco), 1.5 g/L NaHCO_3_ (Cat. # 25080094, Gibco), and 1% NEAA (Non-Essential Amino Acids) (Cat. # 11140050, Gibco). HCT116 cells were cultured in McCoy’s 5a (Cat. # 12330031, Gibco) supplemented with 10%FBS. Cells were grown in a 5% CO_2_ incubator at 37°C. All CRC cell lines were obtained from the American Type Culture Collection (ATCC) and initially tested to confirm they were mycoplasma-free.

#### *In vivo* xenograft model

To establish the xenograft tumor model, male BALB/c-nude mice (6–8 weeks old) were randomly assigned to groups (*n* = 5), and 3 × 10^6^ cells were injected subcutaneously into each mouse. Mice were housed in SPF-grade conditions. From day 5 (tumor palpable), mice tumors were measured (length and width with vernier calipers) every 3 days and data were recorded. Tumor volume (mm^3^) was calculated as follows: volume (mm^3^) = (width^2^ × length)/2. The mice were humanely euthanized using Avertin (Sigma-Aldrich, Shanghai, China). Tumors and lungs excised from the mice were fixed in formalin, embedded in paraffin, and sectioned. The animal experiments were approved by Jining Medical University Animal Ethics Committee (Number: JNMC-2023-DW-036).

### Method details

#### Data preparation

The study utilized RNA sequencing data from 521 colorectal cancer cases obtained from TCGA database, which included relevant pathological and clinical information. Additionally, RNA sequencing data and clinical details were gathered from four independent microarray datasets (GSE39582, GSE44076, GSE44861, GSE41258) available in the GEO database.

In the datasets GSE39582, GSE41258, and TCGA, patients diagnosed with colon cancer were stratified into subgroups based on T, N, M classifications, and disease stage. Furthermore, within the TCGA dataset, subgroups were also categorized according to age, gender, and the presence of lymphatic and neural infiltration. The expression levels of the FAM65A gene across these subgroups were analyzed to elucidate its correlation with significant clinical features of colon cancer.

#### Survival prognosis analysis

The study utilized Kaplan-Meier survival analyses on the GSE39582 and GSE41258 datasets to assess the effect of *FAM65A* expression on OS in colon cancer patients. Additionally, analyses on the TCGA dataset evaluated *FAM65A*’s impact on OS (Overall Survival), DSS (Disease-Specific Survival), and PFI (Progression-Free Interval). Multivariate Cox proportional hazards regression analyses were conducted on the GSE39582 and TCGA datasets using the R package “Survival”, and the regression coefficients from the Cox model were used to create column line plots for predicting OS at 1, 3, and 5 years.

#### Functional enrichment and pathway analysis of *FAM65A* in colon cancer

The mRNA sequencing data employed in this study were sourced from the publicly accessible TCGA database (https://cancergenome.nih.gov/). Samples were stratified into high-expression and low-expression cohorts according to the mean expression level of *FAM65A*. Differential gene expression analysis between these groups was conducted using the R package limma (version 3.40.6). The criteria for initial gene selection were set at *p* < 0.01 and a log fold change greater than 1.5. Functional annotation clustering was performed utilizing the DAVID bioinformatics resource (https://david.ncifcrf.gov/) under default settings. To elucidate the molecular mechanisms associated with the enriched genes, analyses including GO annotation, KEGG pathway enrichment, Reactome pathway analysis, and GSEA were conducted.

#### Plasmids construction

Human *FAM65A* shRNA oligos were synthesized, annealed to form double-stranded oligos, and ligated into linearized pLV-shRNA-puro vector (Cat. #B19, Biosettia,USA) to construct circled pLV-shRNAs-Puro. The human *FAM65A* overexpression vector (pLVML-3×FLAG-FAM65A) was obtained from Fenghui Biology Company (Changsha, China). The primers and oligonucleotide sequences are listed in key resources table.

#### Immunohistochemistry

Immunohistochemical analysis was conducted utilizing human colorectal cancer tissue microarrays (cat. #D150Co01, Bioaitech, Xi’an, China) as well as paraffin-embedded specimens derived from mouse xenograft tumors. Target-specific antibodies were employed to detect corresponding proteins, with evaluation criteria based on either the percentage of positively stained cells or the staining intensity within each tissue sample. Images were acquired using a panoramic viewer at magnifications of ×10 or ×40 (3DHistech, Hungary).^27^

#### Western blotting

Total cellular proteins were extracted using RIPA (Radioimmunoprecipitation Assay) buffer (Cat. # 89901, Thermo Scientific). Equal amounts of protein were loaded onto a 4–20% SDS-PAGE gradient precast gel and transferred to nitrocellulose membranes. Membranes were blocked at room temperature with 5% skimmed milk powder for 1 h. Following blocking, membranes were incubated overnight at 4°C with the primary antibody(s) specified in key resources table. Secondary antibody incubation was then performed at room temperature for 1 h. Protein bands were detected using the Pierce ECL Protein Detection Kit (Cat. #E423-02, Vazyme, Nanjing, China) and visualized via an imaging system. All Western Blot experiments comprised three independent biological replicates.

#### Cell counting kit-8 (CCK-8) assay

A total of 5,000 cells were plated in each well of 96-well plates and incubated for varying durations of 0, 12, 24, 48, 72, and 96 h 10 μL of CCK-8 reagent (Cat. # CK04, Dojindo, Japan) was introduced to each well, followed by a 2-h incubation period. The absorbance was subsequently measured at 450 nm using an automated multi-scanner.

#### Colony formation assay

CRC cells were enumerated and subsequently seeded into 12-well plates at a density of 500 cells/mL. The cells were cultured for a period of 10–14 days, after which they were fixed in paraformaldehyde for 10 min. Following fixation, the cells were stained with Giemsa staining solution for 5 h, rinsed twice with PBS (Phosphate-Buffered Saline), dried, and then photographed. The colonies were quantified using Photoshop software.

#### TUNEL apoptosis assay

Cells (1 × 10^5^) were cultured in a 48-well plate until they reached 90% confluence. Apoptosis was assessed using the One Step TUNEL Apoptosis Assay Kit (Cat. #C1089, Beyotime Biotechnology, Shanghai, China), following the manufacturer’s instructions.

#### EdU-488 cell proliferation assay

Cells (5 × 10^5^) were seeded and grown to 90% confluence. The cell proliferation was detected using a EdU-488 Cell Proliferation Assay Kit (Cat. #C0071S, Beyotime Biotechnology, Shanghai, China), following the manufacturer’s instructions.

#### Wound healing assay

Cells (5 × 10^5^) were seeded and grown to 90% confluence. A scratch wound was created using a sterile pipette tip, and images were captured at 0 and 24 h using an inverted microscope (Olympus, Tokyo, Japan).

#### *Trans*-well migration assay

CRC cells (1 × 10^5^) were suspended in 1% serum medium and seeded into Boyden chamber inserts with 8 μm pore membranes (Corning, NY, USA). Medium containing 10% FBS was added to the lower chamber. After 24 h, the inserts were fixed, washed, and stained with 0.1% crystal violet. Images were captured for statistical analysis.

#### Inhibitor assay

The Ras/ERK was inhibited by the selective Ras inhibitor Abd-7 (Cat. # HY-122862, MedChemExpress, USA). CRC cells were exposed to Abd-7 at concentrations of 10 μM for a duration of 24 h, after which additional experiments were conducted. The phosphorylation of RSK was inhibited by the selective RSK family kinase inhibitor BRD7389 (Cat. # HY-12185, MedChemExpress, USA). CRC cells were exposed to BRD7389 at concentrations of 4 μM and 8 μM for a duration of 24 h, after which additional experiments were conducted.

#### Human phosphokinase array

Phosphorylation levels of selected kinases and proteins were measured using a Human Phosphokinase Array Kit (Cat. # ARY003C, R&D Systems, USA), following the manufacturer’s instructions.

#### Immunoprecipitation (IP)

HCT116-FAM65A cells were cultured in a 10 cm dish. Proteins were extracted, and the primary antibody was incubated with the lysate overnight at 4°C. Protein A/G PLUS-agarose (Cat. # A3124, Santa Cruz, USA) was added, followed by incubation at 4°C for 2 h and centrifugation. The precipitate was washed twice with RIPA buffer, and loading buffer was added. Western blotting was subsequently performed.^30^

#### Immunofluorescence (IF)

Cells were seeded at a density of 2 × 10^5^ cells per well in a 24-well plate. Following culture, the cells were rinsed with PBS and subsequently fixed using 4% paraformaldehyde for 15 min at ambient temperature. Permeabilization was conducted with 0.5% Triton X-100 for 15 min, after which cells were blocked with 5% goat serum for 1 h. The samples were then incubated with the primary antibody overnight, followed by incubation with the corresponding secondary antibody for 1 h at room temperature. Nuclear staining was performed using DAPI, and fluorescence was observed using a confocal microscope. Detailed information regarding the primary and fluorescent secondary antibodies employed is provided in key resources table.

### Quantification and statistical analysis

#### Statistical analysis

Data were analyzed using GraphPad Prism10.0 software (La Jolla, CA, USA). Values are expressed as the mean ± SEM. P-values were calculated using a two-tailed Student’s *t* test (two groups) or one-way ANOVA (more than two groups) unless otherwise noted. A value of *p* < 0.05 was used as the criterion for statistical significance. ∗Indicates significant difference with *p* < 0.05, ∗∗indicates significant difference with *p* < 0.01, ∗∗∗indicates significant difference with *p* < 0.001.

## Acknowledgments

This work was supported by the Shandong Provincial Natural Science Foundation (no. ZR2021QH113 to Y.H.), 10.13039/501100001809National Natural Science Foundation of China (no. 82173192 to W.S.), Shandong Provincial Natural Science Foundation (no. ZR2023YQ067 to W.S. and no. ZR2023QH082 to X.Z.), PhD research foundation of affiliated hospital of Jining Medical University (no. 2022-yxyc-001 to Y.H.), Foundation of Shandong Medical and Health Science and Technology project (no. 202302080890 to J.Y.), and nursery fund of affiliated hospital of Jining Medical University (no. MP-MS-2023–13 to Y.S.).

## Author contributions

Y.M. and J.Y. designed the experiments; X.S., S.W., X.Y., and Y.L. prepared the materials and performed the experiments; Y.S. summarized and analyzed the data; G.T. and X.Z. helped to collect the CRC tumor tissue samples; W.S. and Y.H. wrote the manuscript and reviewed and revised the manuscript.

## Declaration of interests

The authors declare no competing interest.
