# Supplementary material for: FAM65A, as a potential predictor of prognosis, promotes colorectal cancer progression via activating Ras/ERK/RSK signaling
Source: iScience. 2026 Jan 10;29(2):114662. doi: 10.1016/j.isci.2026.114662 (PMC12874459; doi:10.1016/j.isci.2026.114662)
Supplement: Document S1. Figures S1 and S2 and Table S1 [file mmc1.pdf]

iScience, Volume *29*

Supplemental information

FAM65A, as a potential predictor of prognosis, promotes colorectal cancer progression via activating Ras/ERK/RSK signaling

Yuqiu Ma, Jie Yao, Xinzhuang Shen, Shuying Wang, Gongli Tang, Xiaowen Yang, Yifei Li, Yifang Sun, Wenzhi Shen, Xiaoyuan Zhang, and Yongming Huang

Figure S1

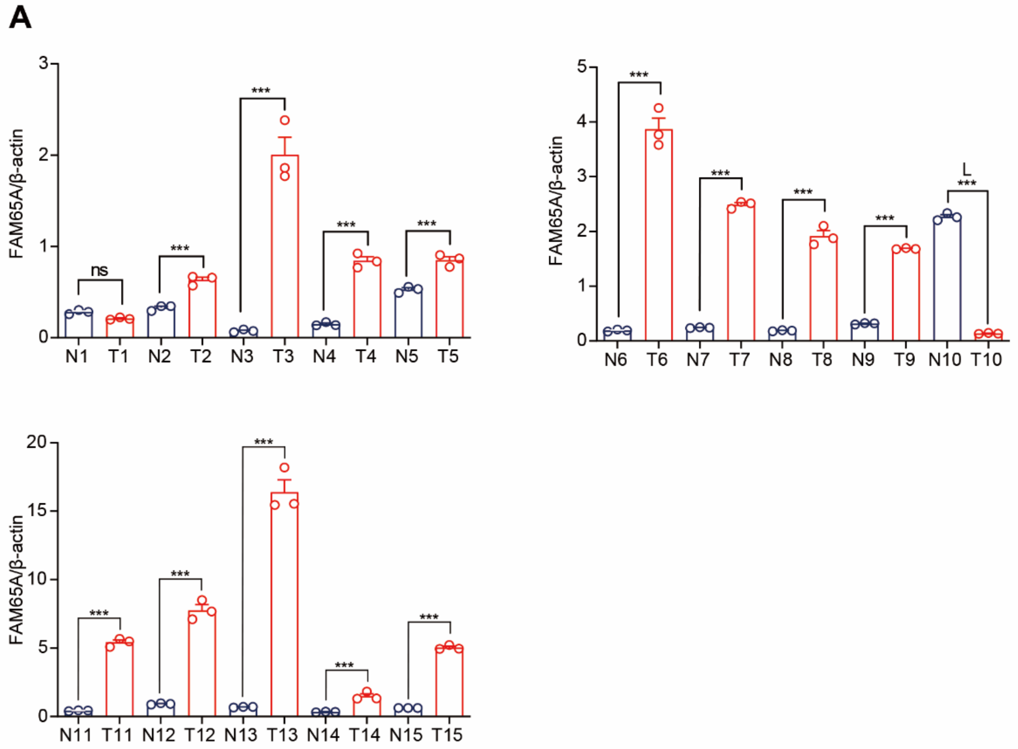

Figure S1. Quantification results of FAM65A expression in the CRC tissue array were shown.

Figure S2

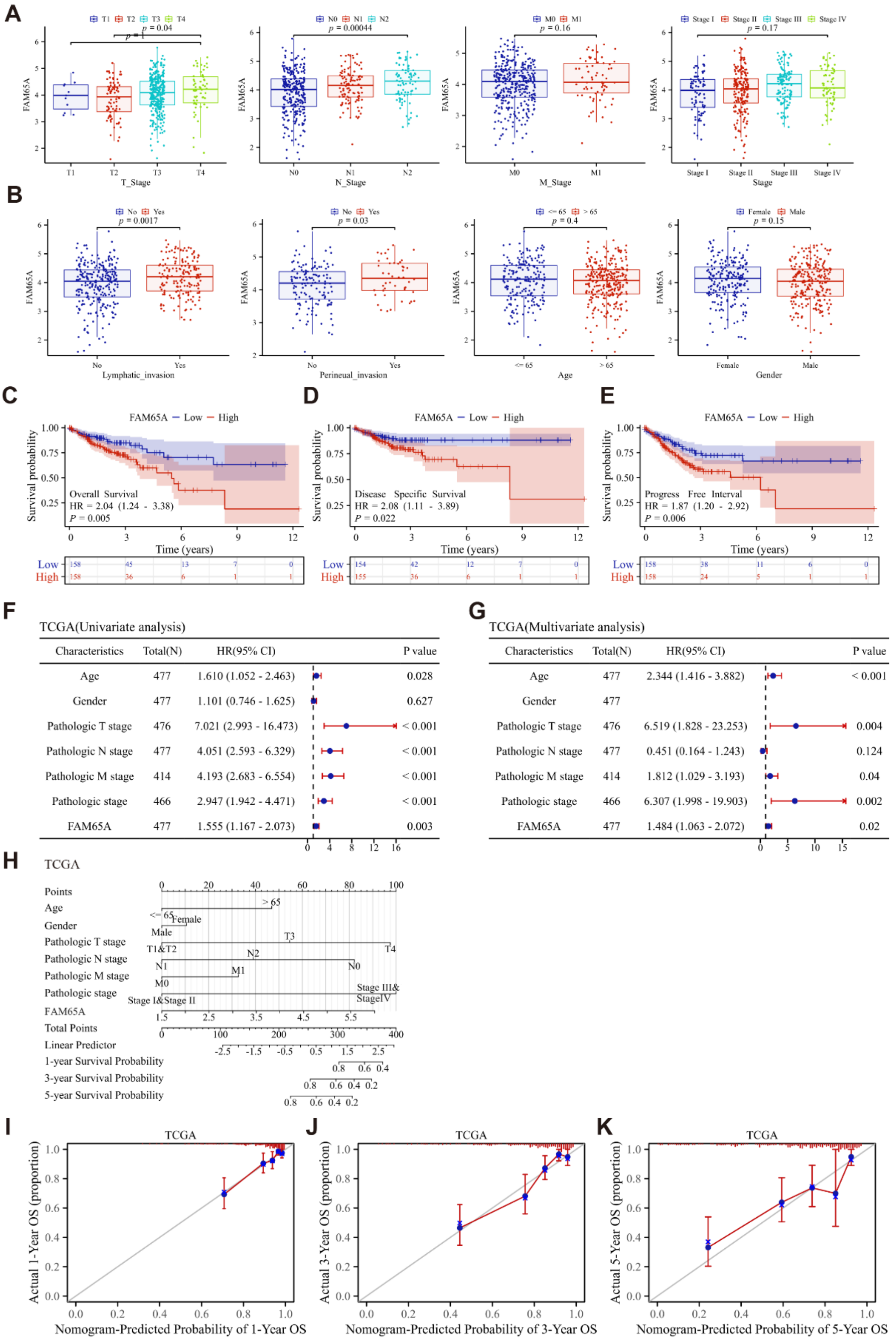

Figure S2. FAM65A as an Independent Prognostic Biomarker Correlated with Clinicopathological Features in CRC Patients

A & B. The clinicopathological correlation between FAM65A expression and colon

cancer in the TCGA database. **C-E**. Prognostic relationship between FAM65A and colon cancer patients in the TCGA database, OS, DSS and PFI. **F & G**. The results of both univariate and multivariate Cox regression analyses conducted on the TCGA dataset. **H**. The construction of a nomogram model incorporating FAM65A within TCGA dataset. **I-K**. The calibration curves for the 1-, 3-, and 5-year nomograms.

Table S1. Patients information

Sample Number	Sex	Age	Organ	Pathology Diagnosis	Type	Sampling Time	Blood
#1	M	63	Colon	Adenocarcinoma	Malignant	16th Jul, 2018	No
#2	M	62	Colon	Adenocarcinoma	Malignant	27th Aug, 2018	No
#3	M	64	Colon	Adenocarcinoma	Malignant	31th May, 2017	No
#4	F	67	Colon	Adenocarcinoma	Malignant	19th Mar, 2018	No
#5	M	62	Colon	Adenocarcinoma	Malignant	2th Jul, 2018	No
#6	M	64	Colon	Adenocarcinoma	Malignant	30th Aug, 2018	No
#7	M	49	Colon	Adenocarcinoma	Malignant	20th Sep, 2018	No
#8	M	64	Colon	Adenocarcinoma	Malignant	23th Jan, 2019	No
#9	F	62	Colon	Adenocarcinoma	Malignant	8th Dec, 2018	No
#10	M	74	Colon	Adenocarcinoma	Malignant	6th Sep, 2018	No
#11	M	47	Colon	Adenocarcinoma	Malignant	18th Oct, 2018	No
#12	M	75	Colon	Adenocarcinoma	Malignant	6th Sep, 2018	No
#13	M	50	Colon	Adenocarcinoma	Malignant	4th Oct, 2017	No
#14	M	42	Colon	Adenocarcinoma	Malignant	13th Oct, 2018	No
#15	M	69	Colon	Adenocarcinoma	Malignant	27th Oct, 2018	No
